# Crystal Structure of *Legionella* DotD: Insights into the Relationship between Type IVB and Type II/III Secretion Systems

**DOI:** 10.1371/journal.ppat.1001129

**Published:** 2010-10-07

**Authors:** Noboru Nakano, Tomoko Kubori, Miki Kinoshita, Katsumi Imada, Hiroki Nagai

**Affiliations:** 1 Research Institute for Microbial Diseases, Osaka University, Suita, Osaka, Japan; 2 Graduate School of Frontier Biosciences, Osaka University, Suita, Osaka, Japan; The Rockefeller University, United States of America

## Abstract

The Dot/Icm type IVB secretion system (T4BSS) is a pivotal determinant of *Legionella pneumophila* pathogenesis. *L. pneumophila* translocate more than 100 effector proteins into host cytoplasm using Dot/Icm T4BSS, modulating host cellular functions to establish a replicative niche within host cells. The T4BSS core complex spanning the inner and outer membranes is thought to be made up of at least five proteins: DotC, DotD, DotF, DotG and DotH. DotH is the outer membrane protein; its targeting depends on lipoproteins DotC and DotD. However, the core complex structure and assembly mechanism are still unknown. Here, we report the crystal structure of DotD at 2.0 Å resolution. The structure of DotD is distinct from that of VirB7, the outer membrane lipoprotein of the type IVA secretion system. In contrast, the C-terminal domain of DotD is remarkably similar to the N-terminal subdomain of secretins, the integral outer membrane proteins that form substrate conduits for the type II and the type III secretion systems (T2SS and T3SS). A short β-segment in the otherwise disordered N-terminal region, located on the hydrophobic cleft of the C-terminal domain, is essential for outer membrane targeting of DotH and Dot/Icm T4BSS core complex formation. These findings uncover an intriguing link between T4BSS and T2SS/T3SS.

## Author Summary

Bacterial pathogens deliver virulence factors such as exotoxins and effector proteins to host cells. To accomplish this bacteria utilize specialized secretion systems such as type III and type IV secretion systems. The type IV secretion systems (T4SS) play a central role in pathogenesis by many important pathogens including *Agrobacterium tumefaciens*, *Helicobacter pylori* and *Legionella pneumophila.* T4SS is ancestrally related to the bacterial conjugation system and is divided into two subgroups, type IVA (T4ASS) and type IVB (T4BSS), which are derived from distinct conjugation systems. In spite of its pivotal role in bacterial pathogenesis, the structural bases and molecular mechanisms of the type IVB secretion still remain largely unknown. Here we show the crystal structure of DotD, one of the core components of *Legionella* T4BSS. Surprisingly, the structure of DotD is not related to those of T4ASS core components. In contrast, the structure of DotD is remarkably similar to that of a subdomain of secretin family proteins, which form substrate conduits for other types of secretion systems. This finding provides intriguing insights into the nature and the evolution of bacterial secretion systems essential for pathogenesis.

## Introduction

Pathogenic bacteria transport functional proteins, such as effector proteins and exotoxins, across bacterial membranes. These bacterial proteins interact with host proteins to manipulate host cellular functions. Therefore, the secretion process plays a central role in bacterial pathogenesis. To accomplish this, bacteria have evolved various secretion systems. The type II secretion system (T2SS) is specialized to export periplasmic protein substrates, such as cholera toxin of *Vibrio cholerae* and heat-labile enterotoxin of enterotoxigenic *Escherichia coli* (ETEC), across outer bacterial membranes to the extracellular milieu [1], [2], [3], [4], [5]. The type III secretion system (T3SS) is a protein-transport mechanism that translocates cytoplasmic substrates directly into the host cytoplasm. It plays a critical role in pathogenesis for a number of important bacterial pathogens, including enteropathogenic *E. coli* (EPEC) [6]. T3SS is ancestrally related to the bacterial flagellar system, and its core apparatus has a characteristic structure, often referred as “needle complex” [7], [8]. The type IV secretion system (T4SS) is related to the conjugation system, and is apparently a very versatile secretion system for biological macromolecules [9], [10]. For example, the *Agrobacterium tumefaciens* VirB/VirD system, one of the best studied T4SSs, is able to transport DNA-protein complex (T-DNA) into host cells. *Bordetella pertussis* secretes periplasmic pertussis holotoxin across outer membrane via the Ptl T4SS [11], [12]. Many intracellular pathogens, including *Legionella pneumophila*, translocate a large array of effector proteins to the host cytosol using T4SSs [13], [14].

T4SSs are further divided into two subgroups, type IVA (T4ASS) and type IVB (T4BSS) [15], [16]. These two subgroups of T4SS are not related to each other at sequence level—with some exceptions, including secretion ATPases VirB11/DotB [17], [18], [19]. T4ASS is related to the conjugation systems of plasmids RP4, R388 and pKM101, and found in a number of bacterial pathogens, including plant pathogen *A. tumefaciens*. T4BSS is related to the conjugation systems of plasmids ColIb-P9 and R64, and was originally found in human pathogen *L. pneumophila* [19], [20], [21]. *L. pneumophila* are gram-negative bacteria ubiquitous in fresh water and soil environments [22], [23]. *L. pneumophila* infect and replicate within a wide variety of phagocytic eukaryotic cells, ranging from unicellular amoeba to human macrophages. In cases of human infection, *L. pneumophila* infection can result in a severe form of pneumonia known as Legionnaires' disease. Intracellular replication of *L. pneumophila* requires functional Dot/Icm T4BSS, irrespectively of host species [18], [19]. It has been well established that *L. pneumophila* translocate more than 100 effector proteins into host cytoplasm using the Dot/Icm T4BSS [13]. The zoonotic pathogen *Coxiella burnetii* and the anthropod pathogen *Rickettsiella grylli* are some of known closest relatives to *Legionella*, and both carry T4BSSs closely related to that of *Legionella* [21], [24], [25], [26]. A growing body of bacterial genomic information now suggests that over 20 pathogenic and environmental bacteria carry T4BSSs (Figure S1).

Recent studies demonstrated that the pKM101 conjugation system, a T4ASS, has a lantern-shaped core complex composed of three proteins TraN/VirB7, TraO/VirB9 and TraF/VirB10 [27], [28]. This core complex spans both inner and outer membranes, but its structure is different from other double membrane-spanning secretion systems in architecture and composition. No other double membrane-spanning complex has been isolated and characterized from both T4ASS and T4BSS. However, a putative core complex of the Dot/Icm T4BSS has been suggested through a biochemical study of component proteins [29]. The complex is supposed to contain two outer membrane lipoproteins, DotC and DotD [30], two inner membrane-spanning proteins, DotF and DotG, and one outer membrane-associated protein, DotH. In the absence of other components of the Dot/Icm T4BSS DotH remains unassociated with outer membrane, while lipoproteins DotC and DotD are targeted to outer membrane ([29] and Figure S2). The outer membrane targeting of DotH depends on lipoproteins DotC and DotD, presumably in a manner analogous with the function of pilotin in targeting secretin inT2SS [29]: the outer membrane lipoprotein pilotin is required for the stabilization and outer membrane targeting of the secretin [31], [32]. The outer membrane lipoprotein VirB7 of *Agrobacterium* T4ASS forms a heterodimer with a core component, VirB9, and stabilizes several VirB proteins, including VirB9 [33]; these are thought to be initial steps in assembling the T4ASS complex [9]. Lately, it has been demonstrated that the lipidation site cysteine of pKM101 TraN/VirB7 is essential to the outer membrane association of pKM101 T4ASS core complex, suggesting that TraN/VirB7 has pilotin-like function in terms of outer membrane targeting of the core complex [28]. Thus, the outer membrane lipoproteins DotC/DotD of T4BSS, T2SS pilotins and T4ASS VirB7 seem to play some overlapping role in the secretion apparatus assembly, although they are dissimilar in size and amino acid sequences.

**Figure 1:**
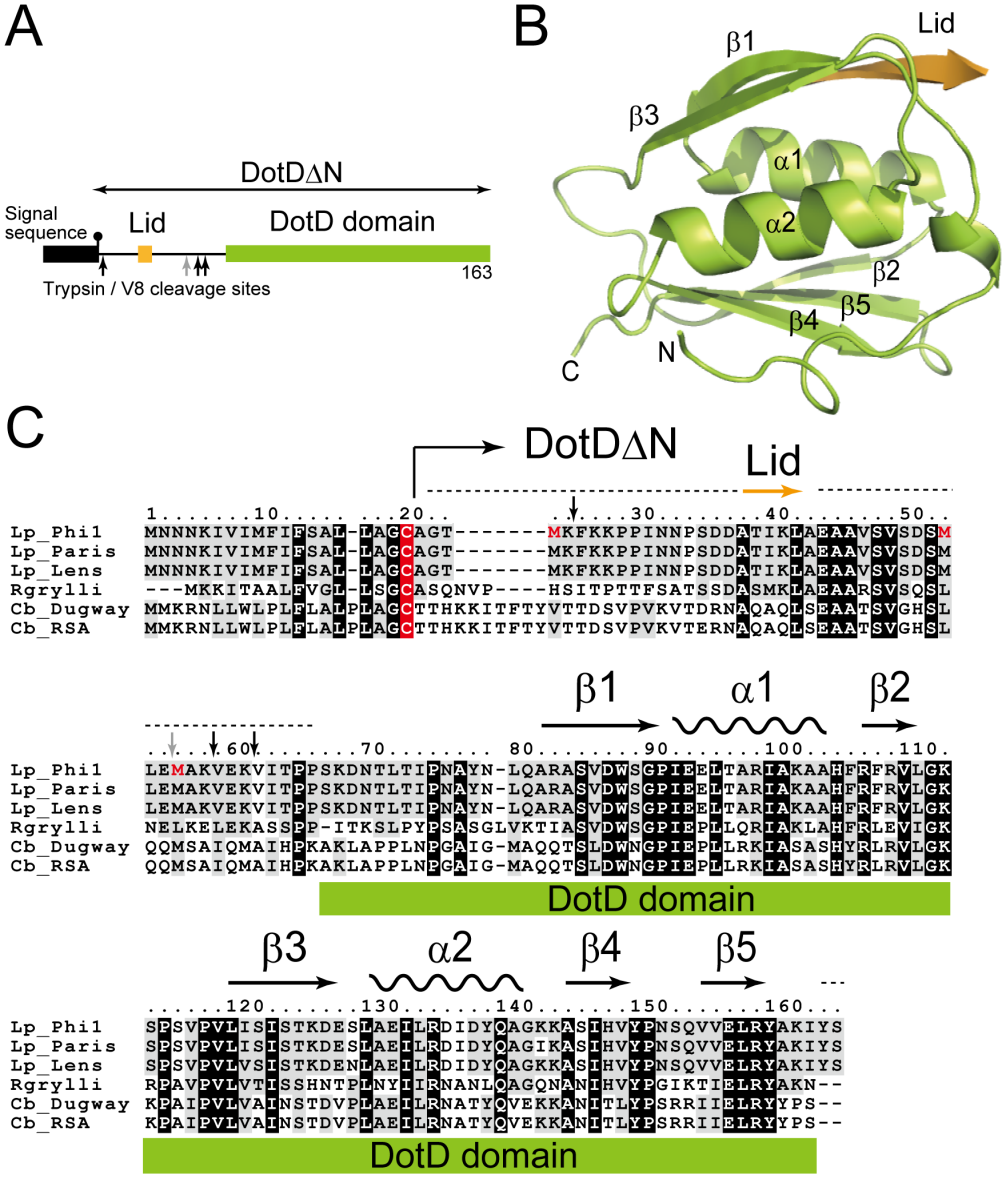
Structure of DotDΔN. (A) Domain structure of DotD. The green and orange boxes denotes the DotD domain and the Lid, respectively, which were visible in the electron density map. The black box denotes the signal sequence. Trypsin and V8 protease preferential cleavage sites are shown by black and grey arrows, respectively. (B) Crystal structure of DotDΔN. (C) Multiple alignment of DotD orthologs from closely related bacterial pathogens, with structural annotation gained from the DotD structure. DotD sequences were obtained from blast nonredundant protein database (nr). Lp: *Legionella pneumophila* (strains Philadelphia-1, Paris, Lens), Rgrylli: *Rickettsiella grylli*, Cb: *Coxiella burnetii* (strains Dugway 5J108-111, RSA 331). Conserved and similar residues were black and grey shaded, respectively. The lipidation site cysteins are red shaded. Tripsin/V8 sites are shows as in panel A.

To clarify the structure and the molecular mechanism of Dot/Icm T4BSS, we crystallized DotD without the first 20 amino acids which contain the signal sequence for secretion across inner membranes and the lipidation site, Cys20 (DotD**Δ**N, Figure 1A), and determined the structure at 2.0 Å resolution.

## Results/Discussion

### Structure of DotDΔN

DotDΔN is composed of the compact C-terminal domain (DotD domain) and the N-terminal disordered region (Figure 1). The N-terminal third of DotDΔN was invisible in the electron density map, except for a short β-strand (Ala-37 to Ala-42) that we call the “lid.” The N-terminal disordering was confirmed with limited digestion experiments using trypsin or V8 protease (Figure S3). Sub-stable products of digestion were identified by mass spectroscopic analysis. All preferential cleavage sites (residues 25, 55, 58 and 61) are found in the N-terminal region, suggesting the N-terminal region is readily accessible to proteases used in this analysis. In addition, all three methionine residues of DotDΔN are in the disordered N-terminal region (shown red letters in Figure 1C). These explain why we were not able to solve the structure by single-wavelength anomalous dispersion (SAD) phasing with the SeMet labeled DotDΔN crystals. Instead, we solved the structure using SAD phasing with Os derivative of DotDΔN crystals.

### Structural similarity between the DotD domain and secretin N0/T3S domains

**Figure 2:**
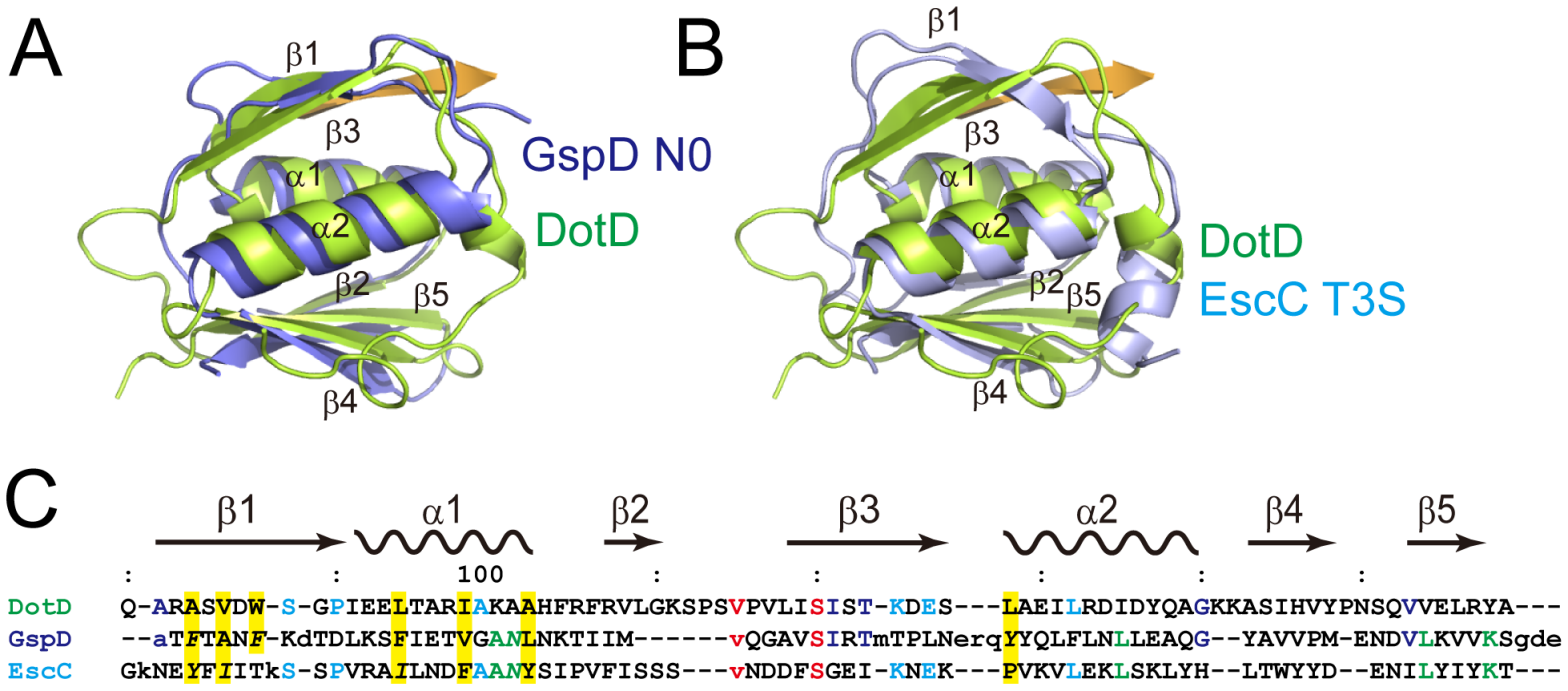
Comparison of DotDΔN with secretin periplasmic subdomains. (A) DotD (green) superimposed onto the N0 domain of ETEC secretin GspD (residue 43 to 120; PDB accession 3ezj) from the type II secretion system (blue). (B) DotD (green) superimposed onto the T3S domain of EPEC secretin EscC (residue 21 to 103; PDB accession 3gr5) from the type III secretion system (light blue). (C) Structure-based sequence alignment of the DotD domain, GspD and EscC. Conserved residues are colored. See text for explanations for yellow shaded residues and residues shown in italics.

The DotD domain forms a βαβ sandwich fold composed of two α-helices flanked by an antiparallel three-stranded β-sheet on one side and a mixed three-stranded β-sheet on the other side (Figure 1B). The DotD domain does not show structural similarity to T4ASS VirB7 [27], [34]. DaliLite database search [35] showed that the DotD domain has striking structural similarity to the N-terminal subdomain of secretins, which are outer membrane pore-forming proteins of T2SS and T3SS (Figure 2). The N0 domain of the ETEC GspD (the protein database (PDB) ID 3ezj, mol-A), a T2SS secretin, is superimposable onto the DotD domain with a root-mean-square deviation (rmsd) of 2.1 Å (Figure 2A). The T3S (type III-specific) domain of the EPEC EscC (PDB ID 3gr5, mol-A), a T3SS secretin, is superimposable onto the DotD domain with a rmsd of 2.5 Å (Figure 2B). Sequence identity between the DotD domain and N0/T3S domains is only 6.4% and 10.6% for 78 and 75 amino acids, respectively. In fact, these domains have never been implicated as a conserved domain at the amino acid sequence level. Thus the DotD domain and the N0/T3S domains of the type II/III secretins share remarkable structural homology, although they are poorly related in amino acid sequences.

### The DotD lid

**Figure 3:**
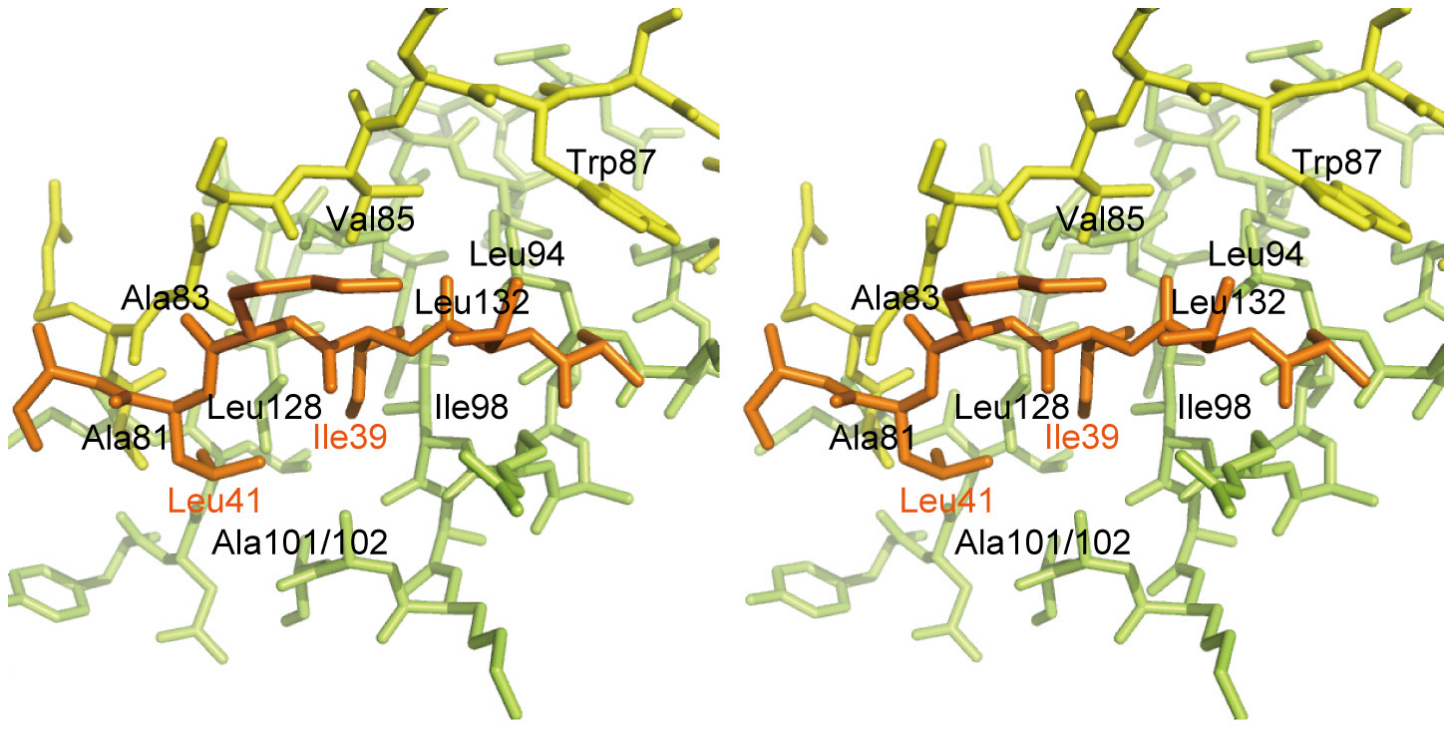
Interaction between the DotD domain and the lid. Stereo view of the interface between the DotD domain (α1 and α2 helices in green, β1 strand in yellow) and the lid (in orange).

The most remarkable difference between DotD and the N0/T3S domains of secretins is that DotD has a lid that makes β-strand addition [36] to β-strands β1 and β3. The lid is located on β1 and covers the hydrophobic cleft formed by α1, α2 and β1 (Figures 3 and S4). The side chains of two hydrophobic residues in the lid (Ile-39 and Leu-41) stick into the cleft. The hydrophobic nature of most secretin residues—corresponding to the DotD residues that form the hydrophobic cleft—is conserved (Figure 2C, yellow shaded). However, some bulky side-chains (Phe-5, Phe-9, Asn-23 and Tyr-51 of GspD, Tyr-32, Ile-34, Ile-44 and Asn-51 of EscC; shown in italics in Figure 2C and in dark blue in Figure S5) protrude inwards and fill the secretin subdomain clefts. As the chain connecting the lid to the DotD domain is invisible in the crystal, it is unclear whether the lid and the DotD domain are in the same molecule.

**Figure 4:**
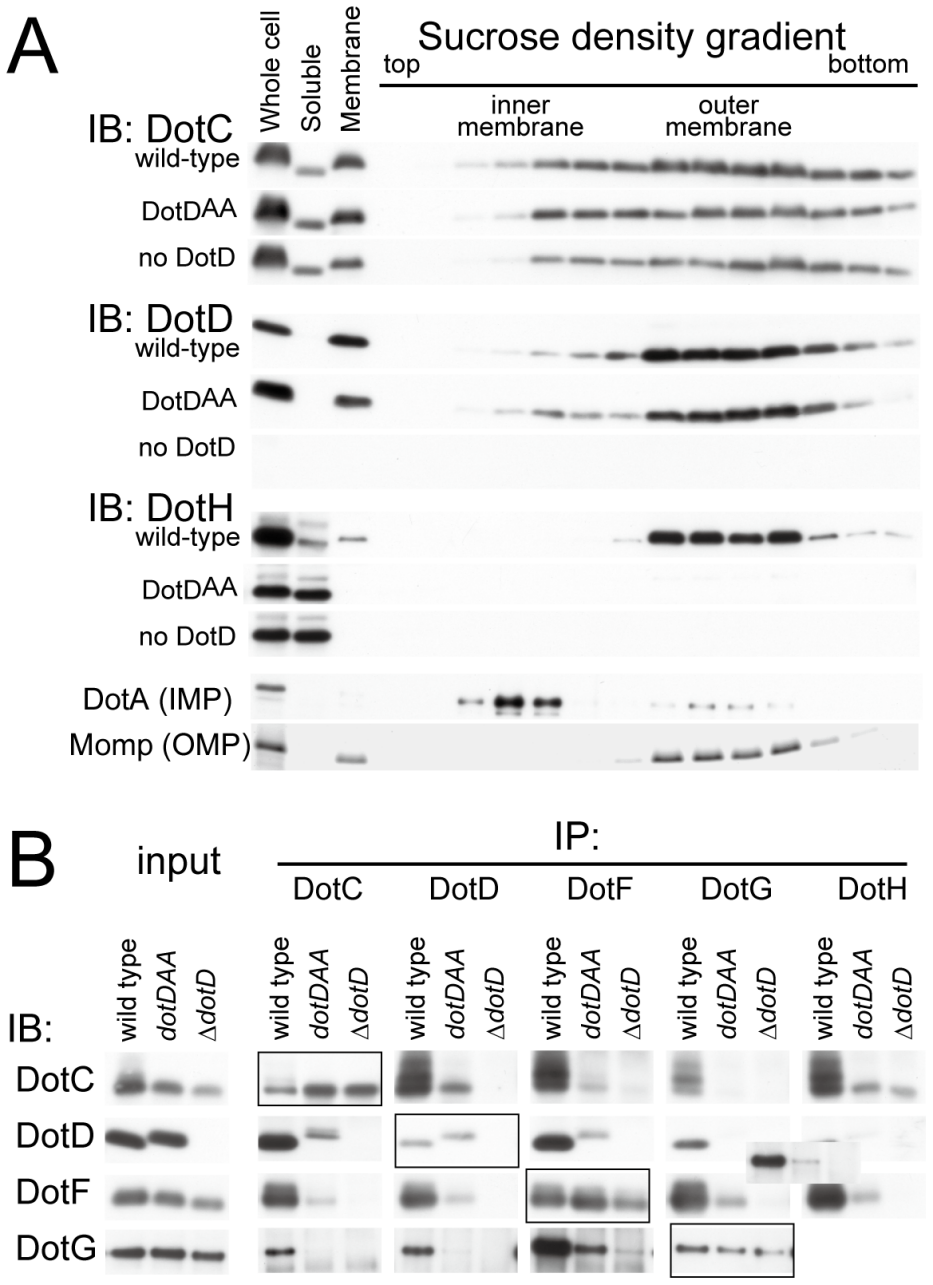
The lid mutation adversely affected core assembly. (A) The lid mutant is defective in outer membrane targeting of DotH. Total membranes were isolated from whole cell lysates of *L. pneumophila* strains producing wild-type, the lid mutant (DotD^AA^) or no DotD. Inner and outer membranes were separated by isopycnic sucrose density gradient centrifugation as described in Materials and Methods. Whole cell lysates (Whole cell), soluble fractions (Soluble), total membranes (Membrane) and the separated membrane fractions were analyzed by Western immunoblotting using indicated antibodies. DotA and Momp were used as inner and outer membrane control, respectively. (B) Adverse effects of the lid mutation on interactions between DotC, DotD, DotF, DotG and DotH. *L. pneumophila* strains producing wild-type, the lid mutant (DotD^AA^) or no DotD were treated with cleavable crosslinker (0.08 mM DSP) before lysate preparation. All *L. pneumophila* strains used in this experiment encode M45 epitope-tagged *dotF* on the chromosome for immunoprecipitation and detection of DotF. Cleared lysates were subjected to immunoprecipitation with antibodies against indicated proteins (IP: DotC, DotD, DotG and DotH) or anti-M45 epitope (IP: DotF) as described in Materials and Methods. Immunoprecipitants were treated with SDS-PAGE sample buffer containing reducing agent to cleave crosslinks, and were subjected to western immunoblotting analyses with antibodies against indicated proteins (IB: DotC, DotD, DotG and DotH) or anti-M45 epitope (IB: DotF). Boxed panels (IP:DotC/IB:DotC, IP:DotD/IB:DotD IP:DotF/IB:DotF, and IP:DotG/IB:DotG) show efficiency of immunoprecitation in this experimental condition. The loading amounts onto the SDS gels of these samples were reduced by 10-fold compared to other samples which show the efficiency of co-immunoprecipitation. Detection of DotH by immunoprecipitation followed by western immunoblotting was technically difficult because its mobility in the gel was similar to that of immunoglobulin heavy chain, and thus was not carried out.

The 16 amino-acid segment (Ala-37 to Met-52) containing the lid is well conserved among DotD orthologs from closely related bacteria (50% identity, Figure 1C), compared with other segments in the N-terminal region of DotDΔN. To evaluate the biological function of the lid, we constructed a lid mutant (DotD^AA^), carrying alanine substitutions of both Ile-39 and Leu-41 residues, the side chains which stick into the cleft. The mutant protein was expressed at a level similar to that of wild-type protein in culture-grown *L. pneumophila*, and was equally targeted to bacterial outer membrane (Figure 4A). Similarly, the expression and the localization of another lipoprotein, DotC, were not affected by the DotD mutation (Figure 4A). In contrast, outer membrane targeting of DotH was abrogated in *L. pneumophila* producing the DotD^AA^ mutant as much as in the DotD deletion strain (Figure 4A). *L. pneumophila* strains producing single mutants DotD^I39A^ or DotD^L41A^ behaved like isogenic wild-type strain (Figure S6), suggesting that alanine substitutions of both residues are required for the defect. Furthermore, immunoprecipitation analyses indicate that all interactions between putative core components DotC, DotD, DotF, DotG and DotH were severely impaired in the *L. pneumophila* producing the mutant DotD^AA^ (Figure 4B). It should be noted that the apparent difference in mobility in SDS gel between the wild-type and the mutant DotD proteins is due to the intrinsic property of these proteins, because the purified mutant protein from overexpressing *E. coli* showed the same anomaly in the gel motility.

These data indicate that the lid plays a significant role in the assembly process of the core complex of the Dot/Icm T4BSS. However, it remains unclear whether the lid functions directly through interaction with the DotD domain, or with other partners such as DotC and DotH, or indirectly through opening the cleft in the DotD domain.

### Periplasmic ring models of DotD

**Figure 5:**
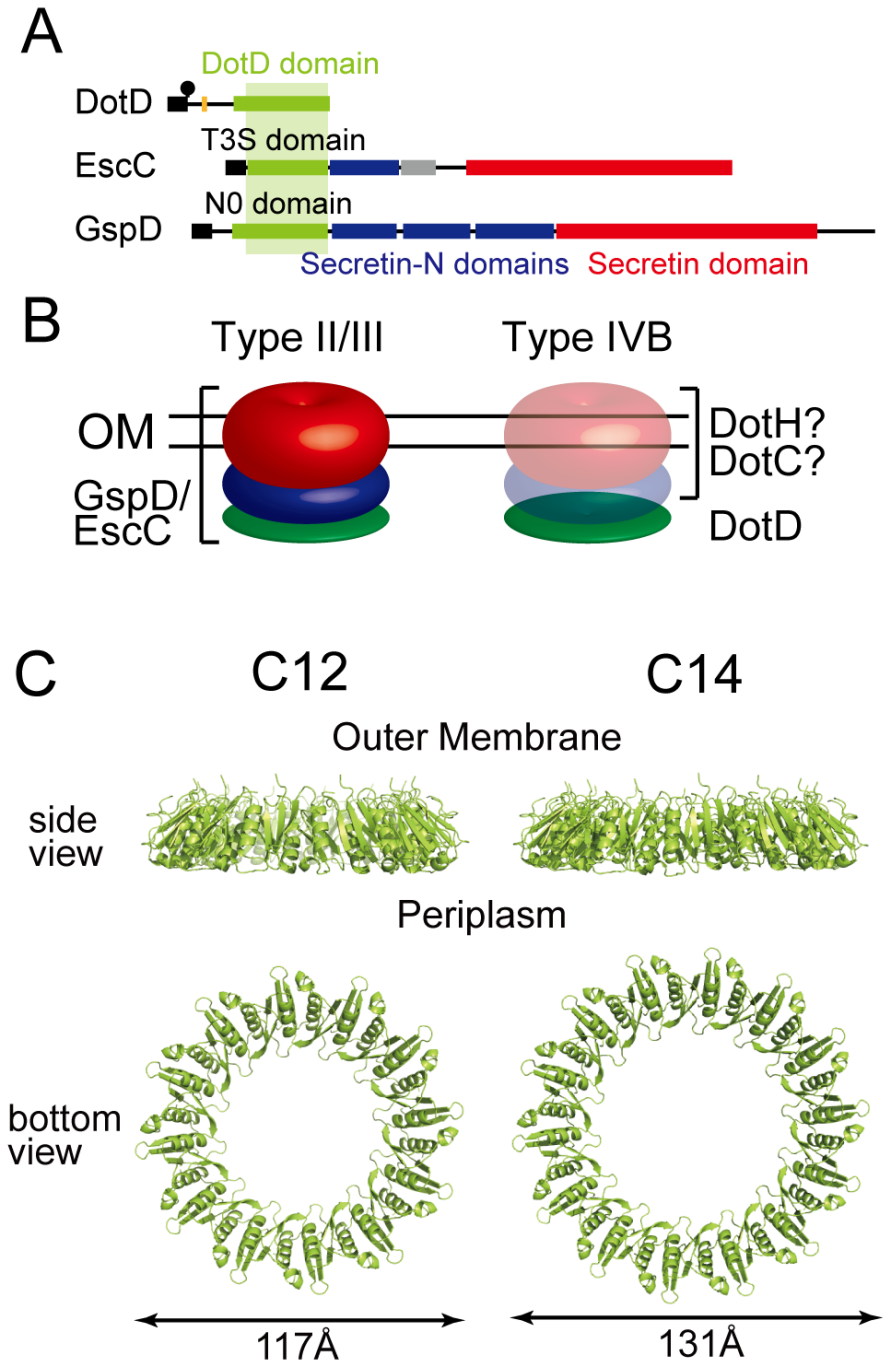
Periplasmic ring models of DotD. (A) Domain organizations of DotD, EscC and GspD. Green boxes: DotD/T3S/N0 domain; Blue boxes: Secretin_N domain (protein family database Pfam PF03958); Red boxes: Secretin domain (Pfam PF00263). (B) Schematic drawings of the type II/III secretin and a putative outer membrane complex containing DotD. Red, blue and green torus represents domains schematically drawn in panel A. (C) Ring models of the DotD domain having 12- and 14-fold rotation symmetry. Ring structures were modeled using SymmDock program [47] fed with DotD atomic coordinates (excluding water molecules and the lid) and order of rotation symmetry (C12 or C14).

Secretins form a protein family that participates in several macromolecule translocation processes across bacterial outer membranes [37], [38], including type II and type III secretion, type IV pilus biogenesis and filamentous phage extrusion. Secretins extracted from membranes are multimeric and have a stacked-ring structure of cylindrical shape. Cryo-electron microscopic analyses of various secretins suggest that secretin rings have 12- or 14-fold rotational symmetry [39], [40], [41]. Their protease-resistant C-terminal domains contain single well-conserved secretin domains (red boxes in Figure 5A), which embed in bacterial outer membranes. The N-terminal region of secretins extends into the periplasm and may interact with inner membrane partner proteins as well as substrates [42], [43]. The N-terminal region is less conserved and always contains one N-terminal domain, which is related to the TonB-dependent outer membrane receptor domain, followed by one or more repeats of domains with the so-called “KH-fold” (green and blue boxes in Figure 5A). The N0/T3S domains are the secretin subdomain closest to the N-terminal that follow the signal sequences for secretion across inner membranes via the Sec machinery. Crystal structures of the periplasmic regions of GspD [44] and EscC [45] secretins, spanning the N0/T3S and the Secretin_N domains, were not captured as multimers of cylindrical shape. However, taken together with available electron micrographic data, it has been suggested that these domains of secretins form periplasmic rings underneath the outer membrane rings. These findings imply that the DotD domain may form a periplasmic ring that is a part of a higher order complex spanning the outer membrane, plausibly composed of DotC, DotD and DotH (Figure 5B).

Our attempts to biochemically isolate a complex containing DotD has not succeeded yet, whereas it is possible to explore the propensity of DotD to form ring using DotD atomic coordinates obtained by this study and an in silico approach. To this end we used SymmDock, an algorithm for prediction of complexes with rotation symmetry by geometry based docking [46], [47] Similar approach using SymmDock has been used for constructing the ring model for GspD periplasmic domain[44]. SymmDock predicted reasonable ring structures of the DotD domain (without the lid), irrespective of the assumption of rotation symmetry, C12 or C14 (Figure 5C and 5D, respectively). These ring models look alike in terms of monomer arrangement in the complexes. The analysis of amino acid conservation pattern using the ConSurf server [48] showed that many conserved residues of both ring models are found at the inner surface and the monomer interface of the DotD domain, while most variable residues are found at the outer surface of the ring (Figure S7). The cleft, the lid-associating site of the DotD domain, comes near the periplasmic surface in the ring models (Figure S8). Therefore, it may affect the interactions between DotD and inner membrane components of the Dot/Icm T4BSS or its substrates. Alternatively, the lid may not be in place and extend towards the outer membrane when the ring is formed. This would be a reasonable possibility given that the N-terminal residue of the DotD domain (Ser-66) is situated at the outer membrane surface of the ring models and the N-terminus of DotD must be anchored in the outer membrane. The crystal structure of the outer membrane complex of the pKM101 T4ASS, containing full-length TraN/VirB7, was recently reported [27]. The TraN/VirB7 in the complex takes an extended conformation and wraps around the outer membrane complex. Mature TraN/VirB7 is a small peptide, 33 residues long, comparable in size to the N-terminal disordered region of DotD (46 amino acids). Along these lines, the N-terminal disordered region of DotD, which contains the lid and the conserved segment (Ala-37 to Met-52), may interact with outer membrane components such as DotC and DotH.

In summary the DotD domain might have the propensity to form a ring like the EscC T3S and the GspD N0 domains; however, the ring models must be validated by future experimental confirmation.

### Links between T2SS and T4BSS

There are several intriguing parallels between secretins and DotC, DotD and DotH, aside from being outer membrane components essential for bacterial secretion systems. The protease-resistant C-terminal domain, representing about two thirds of DotH, is predicted to be rich in β strands using PHDsec [49] (NN and HN, unpublished), which is commonly true of integral outer membrane proteins such as secretins. Lipoproteins DotC and DotD are required for the outer membrane targeting of DotH; likewise pilotins are required for the outer membrane targeting of cognate secretins. Together with the remarkable structural similarity between DotD and a periplasmic subdomain of secretins, it is possible that the putative complex of DotC, DotD and DotH is a secretin counterpart of T4BSS (Figure 5B).

Moreover, there is another link between the secretion ATPases of T4BSS and T2SS. Unlike other T4BSS components, secretion ATPase DotB shares sequence-level similarity with ATPases of T2SS, T4ASS, and the type IV pilus biogenesis system (T4PBS) which is closely related to T2SS. Phylogenetic analysis of these ATPases showed DotB to be closely related to the T4PBS ATPase, PilT [17]. In fact, DotB was found in the major group consisting of T2SS and T4PBS ATPases in the phylogenetic tree inferred for secretion ATPases. Importantly, this group is distinct from the major group in which T4ASS ATPases (VirB11) are found. Collectively, the data from structural and phylogenetic analyses raise the rather unexpected possibility that the architecture of T4BSS machinery shares similarity with T2SS to a certain extent, and can be significantly different from T4ASS.

In conclusion, the present study revealed that the structurally conserved DotD/N0/T3S domain is widely spread throughout outer membrane complexes of even distantly related secretion systems, including T2SS, T3SS and T4BSS. Although the DotD ring models must be validated by future experimental studies, the finding raised the possibility that transport machinery of T4BSS may adopt mosaic architectures of T4ASS and T2SS/T3SS. Future elucidation of structures and functions of bacterial secretion apparatus will give new insights into the molecular mechanism of protein transport across membranes—a central process essential for bacterial pathogenesis.

## Materials and Methods

### Bacterial strains, plasmids and antibodies

Bacterial strains and plasmids used in this study are provided in Table S1. *L. pneumophila* strains defective in *dotD* as well as *L. pneumophila* strains carrying the M45 epitope tagged *dotF* or the *dotD* ^AA^ (I39AL41A) mutation were constructed by allelic exchange [50]. Rabbit anti-sera against DotC, DotG, DotH and M45 epitope were raised by immunization of KHL-conjugated synthesized peptides CMDYVKPEAPNVTLLPKTKA (DotC), CWKQVETQVYTEGTEETK (DotG), CYGPNAKSMPTEEGIPPS (DotH), and CDRSRDRLPPFETETRIL (M45), respectively. Polyclonal antibodies were purified from the anti-sera by affinity chromatography using peptide-conjugated SulfoLink resins (Pierce). Antibodies against DotA (mAb2.29) and DotD were described previously [51], [52].

### Protein expression and purification

*E. coli* cells overproducing DotDΔN with a hexa-histidine tag were collected by centrifugation and resuspended with 50 mM Tris-HCl pH 7.5, 50 mM NaCl, 1 mM EDTA containing Complete Protease Inhibitor Cocktail (Roche Diagnostics). Cells were disrupted, centrifuged (30,000×*g*, 20 min), and the soluble fraction was loaded on a SP sepharose column (GE Healthcare). His-tagged DotDΔN was eluted by a step gradient of NaCl in 20 mM Tris-HCl pH 7.5 and was loaded on a HisSelect column (Sigma-Aldrich). His-tagged DotDΔN was eluted by a step gradient of imidazole in 20 mM Tris-HCl pH 7.5, 150 mM NaCl. Peak fractions were pooled and dialyzed against 20 mM Tris-HCl pH 7.5, 200 mM NaCl. After removal of the His-tag by thrombin digestion, DotDΔN was loaded onto a HiLoad Superdex 75 gel filtration column (GE Healthcare). Purified protein was eluted in 20 mM Tris-HCl pH 7.5 and concentrated using a Vivaspin 20 concentrator (Sartrius). Se-Met DotDΔN was purified using the procedure described above.

### Crystallization, data collection and structure determination

Crystals suitable for X-ray analysis were obtained at 4°C using the sitting-drop vapor-diffusion method. I23 crystals of DotDΔN with unit cell dimensions a = b = c = 103.9 Å were grown from drops prepared by mixing 1 µl protein solution (11.2 mg/ml) with 1 µl reservoir solution containing 8% (v/v) PEG 8000 and 0.1 M CHES-NaOH pH 10.0. Crystals of Se–Met-labeled protein grown under the same conditions as the native crystals were also obtained. Crystals were soaked in a solution containing 90% (v/v) of the reservoir solution and 10% (v/v) MPD for a few seconds, and then immediately transferred into liquid nitrogen for freezing. X-ray diffraction data were collected at a synchrotron beamline BL41XU of SPring-8 (Harima, Japan) with the approval of the Japan Synchrotron Radiation Research Institute (JASRI). The data were recorded under nitrogen gas flow at 90 K, and under He gas flow at 40 K, for native and derivative crystals, respectively. The data were processed with MOSFLM [53] and scaled with SCALA [54]. Initially, we tried to solve the structure using the anomalous data of Se-Met derivative crystals, but the anomalous signal was too weak to determine the phase. Therefore, we prepared Os derivative crystals by soaking the crystals into a reservoir solution containing K_2_OsCl_6_ at 50% saturation for four hours. Initial SAD phase was calculated from the anomalous diffraction data of the Os derivative crystal using SOLVE [55]. The phase was improved and extended to 2.0 Å with DM using a native data set. The model was constructed with COOT [56], and was refined to 2.0 Å using program CNS [57]. A 5% fraction of the data was excluded from the data for the R-free calculation. During the refinement process, iterative manual modifications were performed using an “omit map.” The refinement converged to an R factor of 22.5% and a free R factor of 24.7%. The Ramachandran plot indicated that 91.1 % and 8.9 % residues were located in the most favorable and allowed regions, respectively. Data collection and refinement statistics are summarized in Tables 1 and 2.

**Table 1:** Data collection statistics.

	Native	Os-derivative
Space group	*I2*3	*I2*3
Cell dimension, a(Å)	103.86	103.72
Wavelength (Å)	0.97897	1.13958
Resolution (Å)	42.4-2.0 (2.11-2.0)	42.0-2.6 (2.74-2.60)
*R_merge_*	7.2 (35.2)	9.9 (37.1)
*R_ano_*	-	5.5 (11.6)
I/σI	19.9 (5.6)	23.5 (5.7)
Completeness (%)	99.9 (100)	100 (100)
Redundancy	7.1 (7.3)	6 (6)

### Membrane fractionation

Fractionation of *L. pneumophila* membranes was carried out essentially as described [58]. Briefly, 20 ml of bacterial culture grown in AYE for 18 h with starting OD_600_ = 0.1 was centrifuged and bacterial pellets were suspended with 5 ml of 10 mM HEPES pH 7.4, 20% (w/w) sucrose. After the addition of RNaseA (final 10 µg/ml), DNaseI (2 µg/ml) and phenylmethylsulfonyl fluoride (PMSF, 1 mM), cells were lysed by two passages of a chilled French Pressure Cell (Thermo Scientific); unlysed cells were removed by centrifugation (5,000 g, 15 minutes). EDTA was added to the lysate to a final concentration of 5 mM. A crude membrane fraction was obtained by loading 1.6 ml of the bacterial lysate onto a two-step gradient consisting of 0.8 ml of a 60% sucrose cushion and 2.5 ml of 25% sucrose in 10 mM HEPES. The membranes were pelleted for 3.5 h at 40,000 rpm in an SW50.1 rotor at 4°C. A membrane layer visible on top of the 60% sucrose was extracted and diluted to <25% sucrose with 10 mM HEPES. The crude membrane fraction was separated by isopycnic sucrose density gradient centrifugation using a gradient consisting of a 0.5-ml cushion of 60% sucrose and layers of 1 ml of 55% sucrose, 2.4 ml each of 50%, 45%, and 40% sucrose, 1.4 ml of 35% sucrose, and 1 ml of 30% sucrose in 10 mM HEPES. Approximately 1.2 ml of the crude membrane prep was placed on top of the gradient and centrifuged in an SW41 rotor at 37,000 rpm for 16 h at 4°C. Fractions (0.75 ml) were collected and analyzed by western immunoblotting using antibodies against DotC, DotD and DotH. Levels of the 28-kDa Major outer membrane protein (MOMP) and DotA were determined by Coomassie blue staining and western immunoblotting, respectively.

### Immunoprecipitation analysis

Bacterial cells grown as described above in 10 ml AYE medium were washed once with cold PBS and resuspended in 10 ml of PBS. A cleavable crosslinker, dithiobis (succinimidyl propionate) (DSP; final concentration: 0.08 mM) was added to the suspension and incubated for 2 h on ice. The crosslinking reaction was stopped by addition of Tris pH 8.0 (final 120 mM) and cells were resuspended with 5 ml of 50 mM Tris-HCl pH 8.0, 150 mM NaCl, 1 mM EDTA. A suspension was mixed with lysozyme (1.25 mg/ml) and incubated for 30 minutes on ice. After addition of RNaseA, DNaseI and PMSF, a whole cell lysate was prepared as described above. Total proteins in 1 ml of the lysate were precipitated by final 10% TCA. The precipitates were washed three times with acetone, and dissolved in 100 µl of 50 mM Tris-HCl pH 8.0, 1% SDS, 1 mM EDTA. After the heat treatment (100°C for 3 minutes), 30 µl of denatured lysate was diluted with 1 ml of Triton buffer (2% Triton X-100, 50 mM Tris pH 8.0, 150 mM NaCl, 0.1 mM EDTA). Insoluble materials were removed by centrifugation (14,000 rpm, 20 minutes) and the supernatant fraction was transferred to a new tube. Indicated antibody (0.1 µg) was added to a supernatant and incubated at 4°C for overnight with gentle rotation. Protein A resin (GE Health Care, 10 ul of 50% suspension in Triton buffer) was added to the mixture and incubated for further 2 h with rotation. Resins were washed twice with Triton buffer, once with 50 mM Tris-HCl pH 8.0. Immunocomplexes were extracted with 50 µl of sample buffer containing a reducing agent and analyzed by western immunoblotting.

### Accession number

The atomic coordinates have been deposited in the Protein Data Bank, www.pdb.org (PDB ID code 3ADY).

**Table 2:** Refinement statistics.

Resolution range (Å)	36.7-2.0 (2.13-2.00)
No. of reflections working	12,104 (1,990)
No. of reflections test	623 (96)
R_w_ (%)	22.5 (27.1)
R_free_ (%)	24.7 (27.0)
Rms deviation bond length (Å)	0.005
Rms deviation Bond angle (°)	1.1
B-factors	
Protein atoms	37.8
Solvent atoms	59.5
Ramachandran plot (%)	
Most favored	911
Additionally allowed	8.9
Generously allowed	0
Disallowed	0
No. of protein atoms	794
No. of solvent atoms	181

Values in parentheses are for the highest resolution shell.

R_w_ =  Σ|| Fo | - | Fc || / Σ | Fo |, R_free_ =  Σ || Fo | - | Fc || / Σ | Fo |

## Supporting Information

Table S1 Bacterial strains and plasmids used in this study.
(0.04 MB DOC)

Figure S1 Phylogenetic analysis of a type IVB core component DotD/TraH. The evolutionary history was inferred using the Neighbor-Joining method [63]. The bootstrap consensus tree was inferred from 500 replicates [64]; it is taken to represent the evolutionary history of the taxa analyzed [64]. Branches corresponding to partitions reproduced in less than 50% bootstrap replicates are collapsed. The percentages of replicate trees in which the associated taxa clustered together in the bootstrap test (500 replicates) are shown next to the branches [64]. The tree is drawn to scale, with branch lengths in the same units as those of the evolutionary distances used to infer the phylogenetic tree. The evolutionary distances were computed using the Poisson correction method [65], and are in the units of the number of amino acid substitutions per site. All positions containing gaps and missing data were eliminated from the dataset (Complete deletion option). There were a total of 117 positions in the final dataset. Phylogenetic analyses were conducted in MEGA4 [66].
(0.89 MB TIF)

Figure S2 Outer membrane localization of DotD in the absence of other components of the Dot/Icm system. Total membranes were isolated from whole cell lysates of wild-type *L. pneumophila* strain carrying empty vector (*dotD* ^+^/vector), isogenic dotD deletion strain producing DotD in trans (Δ*dotD*/p*dotD*), or isogenic strain lacking whole dot/icm genes but producing DotD *in trans* (Δ*doticm*/p*dotD*). Inner and outer membranes were separated by isopycnic sucrose density gradient centrifugation as described in Materials and Methods. Whole cell lysates (Whole cell), soluble fractions (Soluble), total membranes (Membrane) and membrane fractions separated by the isopycnic sucrose density gradient centrifugation were analyzed by Western immunoblotting using anti-DotD antibodies. Fractions containg inner and outer membranes were designated on the top of panels.
(0.17 MB TIF)

Figure S3 Mapping of DotDΔN preferential cleavage sites by (A) trypsin or (B) V8 protease challenge. Purified DotDΔN was challenged with trypsin or V8 protease over a 180-minute period. Samples were taken at indicated times, and were subjected to SDS-PAGE and to MS analysis to determine substable species.
(0.41 MB TIF)

Figure S4 Electron density map showing the interaction between the DotD domain and the lid. Stereo view of the 2Fo-Fc map with contour level 0.97e/Å^3^ representing the interface between the DotD domain and the lid (shown in sticks in Figure 3) was generated using COOT [56].
(0.66 MB TIF)

Figure S5 Stereo figures showing the cleft surfaces of DotD, GspD and EscC. Bulky side-chains (Phe-5, Phe-9, Asn-23 and Tyr-51 of GspD, Tyr-32, Ile-34, Ile-44 and Asn-51 of EscC) protruding inwards and filling the clefts of secretin subdomains are shown in dark blue.
(0.69 MB TIF)

Figure S6 The lid single mutations (I39A or L41A) did not affect outer membrane targeting of DotH. Membrane fraction using *L. pneumophila* strains producing wild-type DotD or single mutants DotD^I39A^ or DotD^L41A^ was carried out as in Figure 4A.
(0.17 MB TIF)

Figure S7 Sequence conservation patterns projected on the ring models. Sequences of the DotD/TraH family proteins shown in Fig. S1 were multiple-aligned by ClustalW2 [67]. The resulting alignment was used for calculation by the ConSurf server [48]. Conservation patterns are projected on (A) C12 and (B) C14 ring models.
(2.04 MB TIF)

Figure S8 Ring models of DotD with the lid. The DotD domains are shown in green, and the lids are shown in brown.
(0.58 MB TIF)
