# Supplementary material for: Crystal Structure of Legionella DotD: Insights into the Relationship between Type IVB and Type II/III Secretion Systems
Source: PLoS Pathog. 2010 Oct 7;6(10):e1001129. doi: 10.1371/journal.ppat.1001129 (PMC2951367; doi:10.1371/journal.ppat.1001129)
Supplement: Table S1 — Bacterial strains and plasmids used in this study. (0.04 MB DOC) [file ppat.1001129.s001.doc]

Strains Genotype Reference

BL21(DE3) *E. coli* B strain carrying DE3 prophage [59]

Lp01 *L.pneumophila* serogroup 1, strain Lp01 *rpsL* [60]

NH1017 Lp01 Δ*dotD* This study

NH1085 Lp01 *dotD*^AA^ (I39A L41A) This study

NH1001 Lp01 *dotF-*M45 This study

NH1080 Lp01 *dotF-*M45 Δ*dotD* This study

NH1090 Lp01 *dotF-*M45 *dotD*^AA^ (I39A L41A) This study

NH1075 Lp01 Δ*dotD-icmX* Δ*icmT-dotU* This study

Plasimds Properti es Reference

pET15b *E. coli* expression vector Novagen

pNH1244 pET15b- *dotD*ΔN This study

pMMB207 *L.pneumophila* expression vector [61]

pNH1035 pMMB207 encoding DotD This study

pNH1294 pMMB207 encoding DotD^AA^ (I39A L41A) This study

pNH1381 pMMB207 encoding DotD^I39A^ (I39A) This study

pNH1382 pMMB207 encoding DotD^L41A^ (L41A) This study

pSR47S Gene replacement vector [62]

pNH1027 pSR47S carrying Δ*dotD* allele This study

pNH1009 pSR47S carrying *dotF-*M45 allele This study

pNH1305 pSR47S carrying *dotD*^AA^ (I39A L41A) allele This study
