# Supplementary figures and images for: Crystal Structure of Legionella DotD: Insights into the Relationship between Type IVB and Type II/III Secretion Systems
Source: PLoS Pathog. 2010 Oct 7;6(10):e1001129. doi: 10.1371/journal.ppat.1001129 (PMC2951367; doi:10.1371/journal.ppat.1001129)

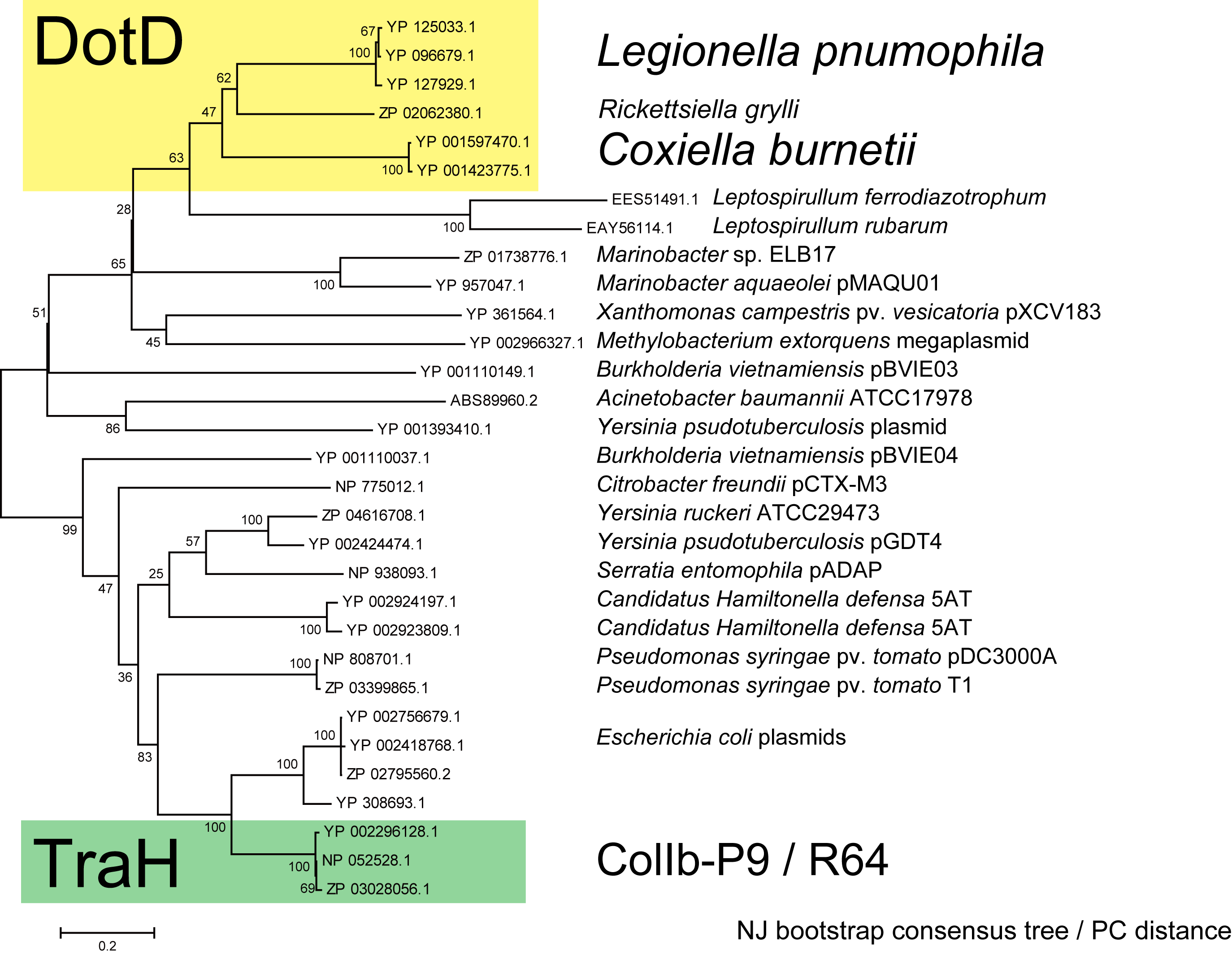

Supplement: Figure S1 — Phylogenetic analysis of a type IVB core component DotD/TraH. The evolutionary history was inferred using the Neighbor-Joining method [63]. The bootstrap consensus tree was inferred from 500 replicates [64]; it is taken to represent the evolutionary history of the taxa analyzed [64]. Branches corresponding to partitions reproduced in less than 50% bootstrap replicates are collapsed. The percentages of replicate trees in which the associated taxa clustered together in the bootstrap test (500 replicates) are shown next to the branches [64]. The tree is drawn to scale, with branch lengths in the same units as those of the evolutionary distances used to infer the phylogenetic tree. The evolutionary distances were computed using the Poisson correction method [65], and are in the units of the number of amino acid substitutions per site. All positions containing gaps and missing data were eliminated from the dataset (Complete deletion option). There were a total of 117 positions in the final dataset. Phylogenetic analyses were conducted in MEGA4 [66]. (0.89 MB TIF) [file ppat.1001129.s002.tif]

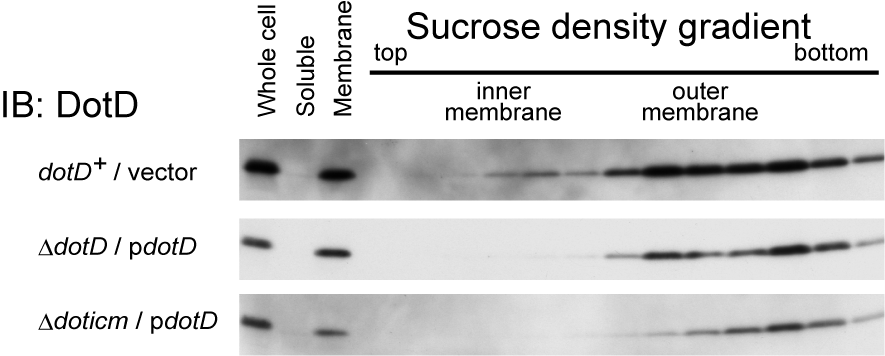

Supplement: Figure S2 — Outer membrane localization of DotD in the absence of other components of the Dot/Icm system. Total membranes were isolated from whole cell lysates of wild-type L. pneumophila strain carrying empty vector (dotD +/vector), isogenic dotD deletion strain producing DotD in trans (ΔdotD/pdotD), or isogenic strain lacking whole dot/icm genes but producing DotD in trans (Δdoticm/pdotD). Inner and outer membranes were separated by isopycnic sucrose density gradient centrifugation as described in Materials and Methods. Whole cell lysates (Whole cell), soluble fractions (Soluble), total membranes (Membrane) and membrane fractions separated by the isopycnic sucrose density gradient centrifugation were analyzed by Western immunoblotting using anti-DotD antibodies. Fractions containg inner and outer membranes were designated on the top of panels. (0.17 MB TIF) [file ppat.1001129.s003.tif]

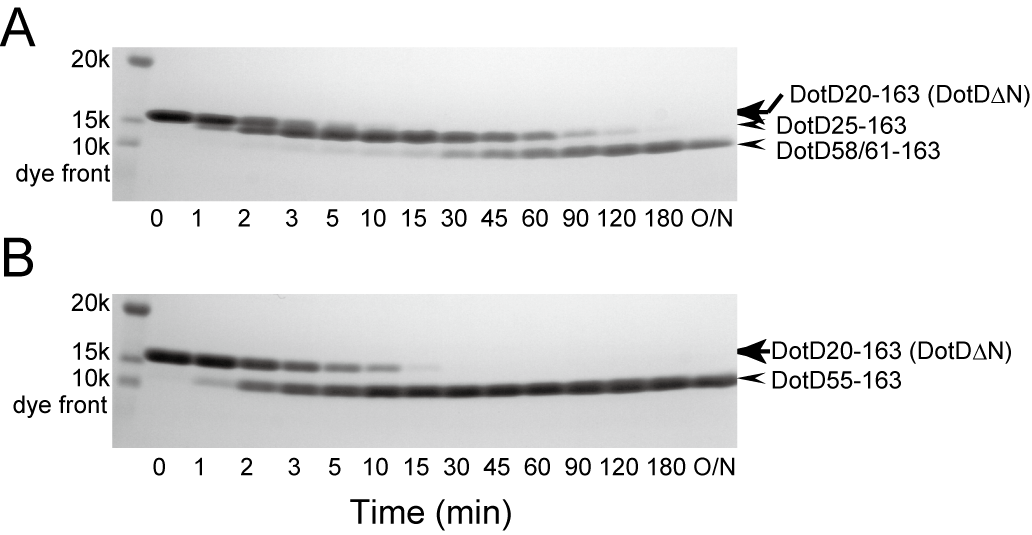

Supplement: Figure S3 — Mapping of DotDΔN preferential cleavage sites by (A) trypsin or (B) V8 protease challenge. Purified DotDΔN was challenged with trypsin or V8 protease over a 180-minute period. Samples were taken at indicated times, and were subjected to SDS-PAGE and to MS analysis to determine substable species. (0.41 MB TIF) [file ppat.1001129.s004.tif]

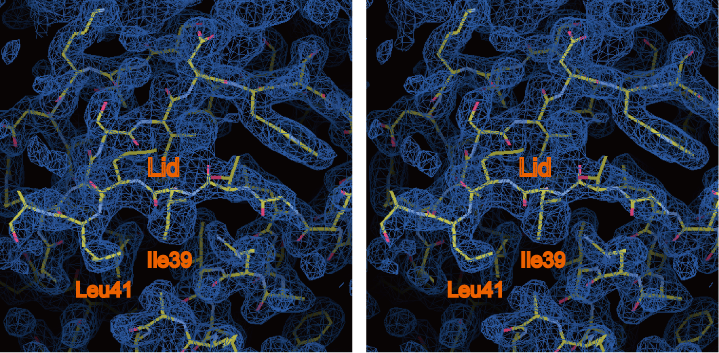

Supplement: Figure S4 — Electron density map showing the interaction between the DotD domain and the lid. Stereo view of the 2Fo-Fc map with contour level 0.97e/Å3 representing the interface between the DotD domain and the lid (shown in sticks in Figure 3) was generated using COOT [56]. (0.66 MB TIF) [file ppat.1001129.s005.tif]

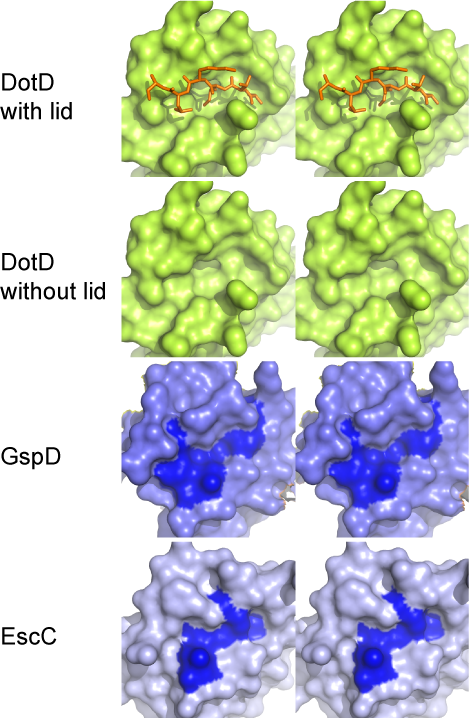

Supplement: Figure S5 — Stereo figures showing the cleft surfaces of DotD, GspD and EscC. Bulky side-chains (Phe-5, Phe-9, Asn-23 and Tyr-51 of GspD, Tyr-32, Ile-34, Ile-44 and Asn-51 of EscC) protruding inwards and filling the clefts of secretin subdomains are shown in dark blue. (0.69 MB TIF) [file ppat.1001129.s006.tif]

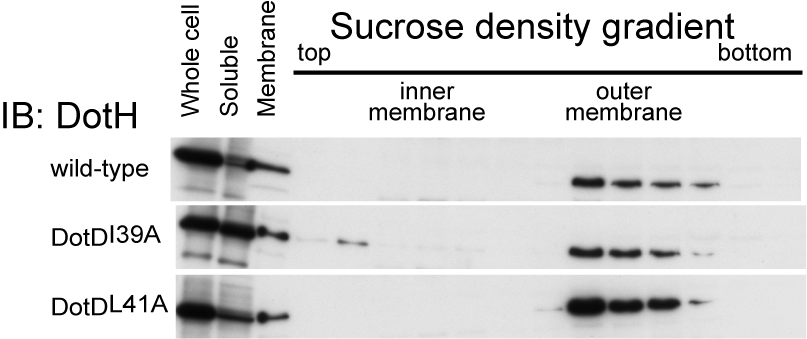

Supplement: Figure S6 — The lid single mutations (I39A or L41A) did not affect outer membrane targeting of DotH. Membrane fraction using L. pneumophila strains producing wild-type DotD or single mutants DotDI39A or DotDL41A was carried out as in Figure 4A. (0.17 MB TIF) [file ppat.1001129.s007.tif]

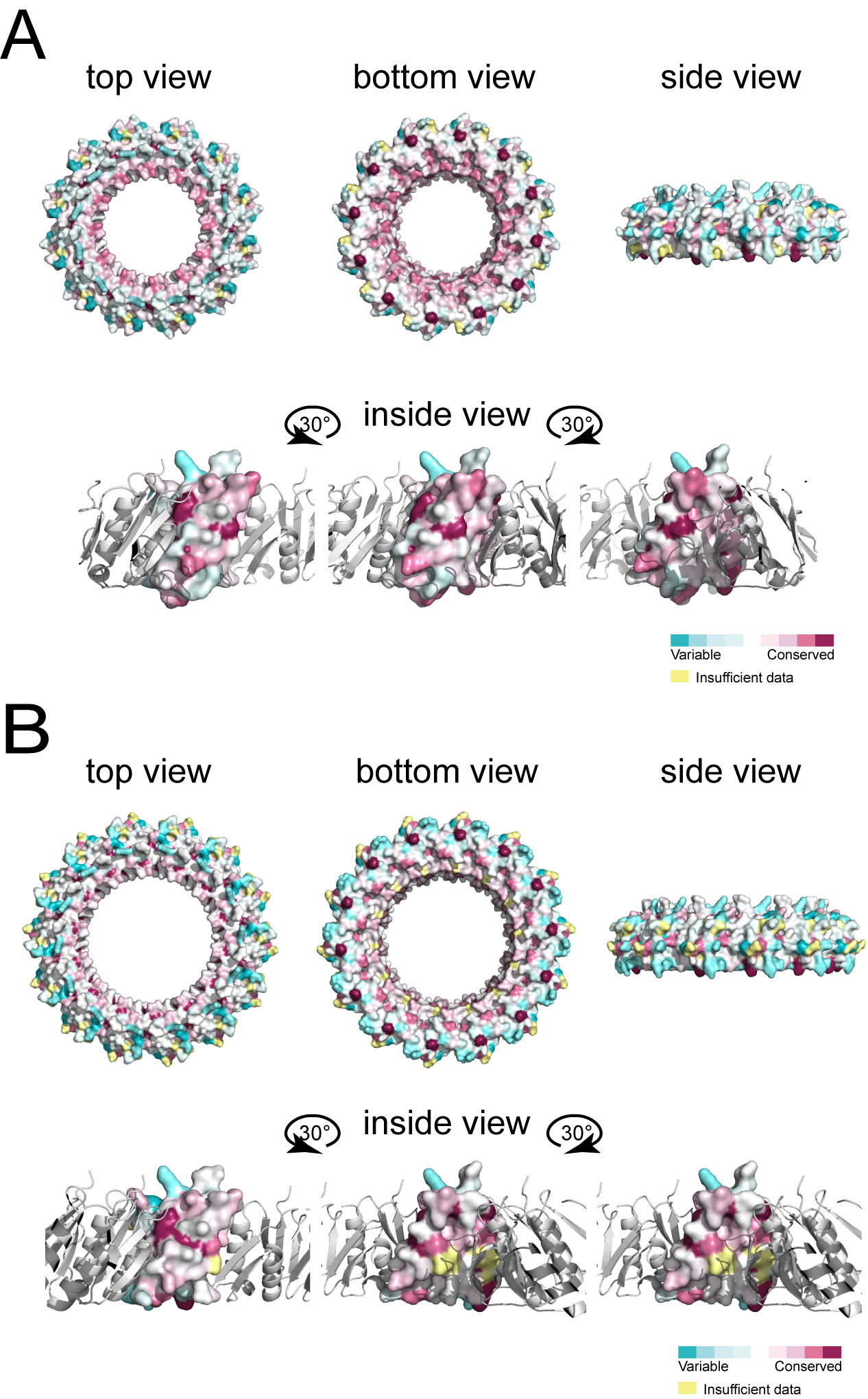

Supplement: Figure S7 — Sequence conservation patterns projected on the ring models. Sequences of the DotD/TraH family proteins shown in Fig. S1 were multiple-aligned by ClustalW2 [67]. The resulting alignment was used for calculation by the ConSurf server [48]. Conservation patterns are projected on (A) C12 and (B) C14 ring models. (2.04 MB TIF) [file ppat.1001129.s008.tif]

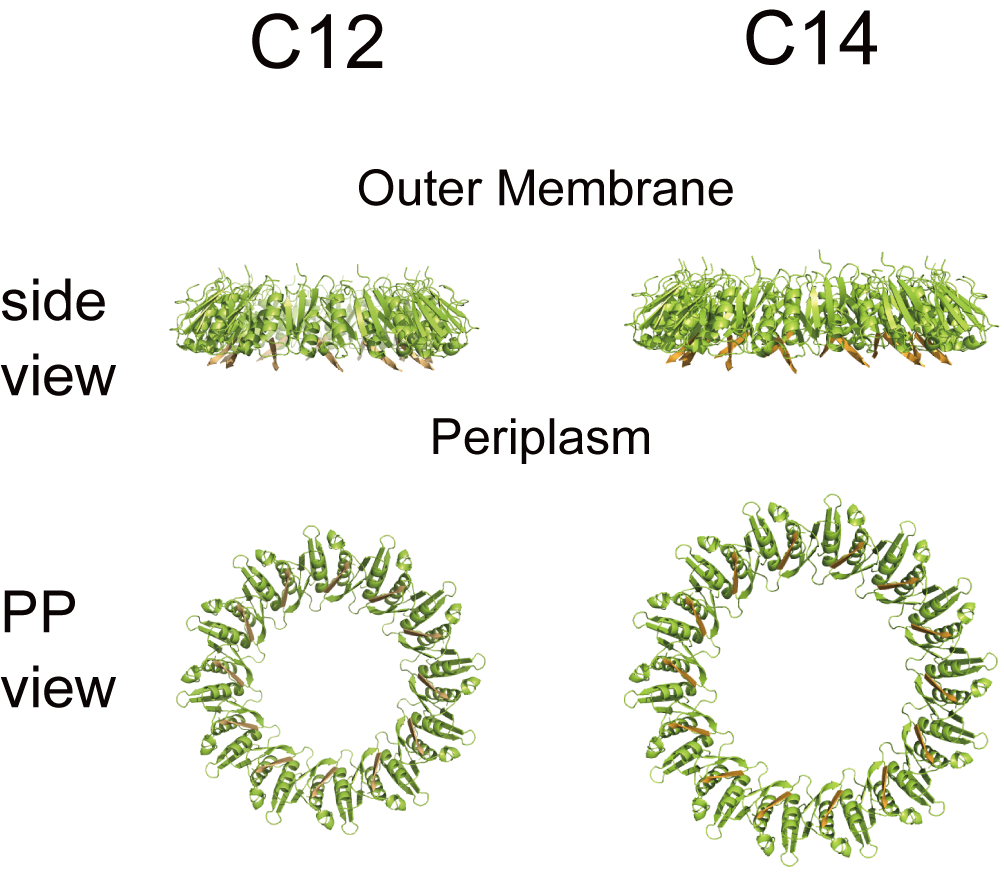

Supplement: Figure S8 — Ring models of DotD with the lid. The DotD domains are shown in green, and the lids are shown in brown. (0.58 MB TIF) [file ppat.1001129.s009.tif]
